# Engineering, Mechanical and Dynamic Properties of Basalt Fiber Reinforced Concrete

**DOI:** 10.3390/ma16020623

**Published:** 2023-01-09

**Authors:** Han Wu, Xia Qin, Xu Huang, Sakdirat Kaewunruen

**Affiliations:** Department of Civil Engineering, School of Engineering, University of Birmingham, Edgbaston B15 2TT, UK

**Keywords:** basalt fiber reinforced concrete, residual flexural strength, modal test, damping ratio, dynamic modulus of elasticity

## Abstract

This study investigates the engineering and mechanical properties of basalt fiber-reinforced (FRF) concrete, giving special attention to residual flexural strength and dynamic modal parameters. These properties, which have not been thoroughly investigated elsewhere, are a precursor to structural design applications for dynamic compliant structures (i.e., bridges, offshore platforms, railways, and airport pavement). Accordingly, the standard notched flexural tests have been carried out to assess the basalt fiber-reinforced concrete’s residual flexural strength with an additional 0.125%, 0.25%, 0.375%, and 0.5% of volume fraction of basalt fiber. In addition, dynamic modal tests were then conducted to determine the dynamic modulus of elasticity (MOE) and damping of the FRF concrete beams. The results indicate that concrete’s toughness and crack resistance performance are significantly improved with added fiber in basalt fiber reinforced concrete, and the optimum fiber content is 0.25%. It also exhibits the highest increment of compressive strength of 4.48% and a dynamic MOE of 13.83%. New insights reveal that although the residual flexural performance gradually improved with the addition of basalt fiber, the damping ratio had an insignificant change.

## 1. Introduction

Fiber-reinforced concrete (FRC) has gained momentum in industrial applications in recent decades, especially for bespoke products and components using a low-to-moderate strength of concrete. With the pioneering publications of steel fiber reinforced in concrete by Romualdi and Batson [1] and Romualdi and Mandel [2], the world has entered a new era of fiber reinforced concrete. The research in fiber reinforced concrete (FRC) has a profound history.

Generally, the improvement of fiber reinforced concrete from ordinary concrete includes flexural strength, spitting strength, compressive strength, elastic modulus, crack resistance, self-healing ability, abrasion resistance, shrinkage and expansion resistance, and thermal characteristics. Meanwhile, with the development of high-performance materials technology, there are more types of fiber, such as glass polypropylene and carbon fiber, that have been applied in the research field of fiber reinforced concrete. The advanced fiber not only contributes to the improvement of mechanical properties, but is more environmental-friendly and even more inexpensive than the traditional steel fiber [2,3,4,5,6,7,8,9,10,11,12,13,14].

Recently, basalt fiber, as one of advanced fibers applied in the field of fiber reinforced concrete, has drawn attention from concrete engineers due to its excellent work in the improvement of mechanical properties and its contribution to sustainable development [15]. However, most previous studies mainly focused on the mechanical properties of basalt fiber reinforced concrete, and the dynamic properties of concrete have not been identified anywhere.

### 1.1. The Mechanical Properties of Basalt Fiber Reinforced Concrete

Basalt is known as a natural, solid, dense, and dark brown to black volcanic igneous rock formed about 100 to 300 km under the Earth’s surface. It can turn the surface into molten lava with a melting temperature ranging from 1500 to 1700 °C [15,16].

The condition of basalt is significantly influenced by the temperature of the quenching process, which results in highly complete crystallization. As the two significant minerals, plagiocene and pyroxene account for over 80% of basalts. By examining the chemical makeup, Fiore V et al. [17] made it feasible to see that SiO_2_ and Al_2_O_3_ are the two primary components.

Basalt fiber is also regarded as a new highly-recommended reinforcement material in concrete compared to the relatively common types, including carbon, aramid, and glass fibers. As the quenching process is environmentally friendly with high security [17,18], such an innovative material has several advantages, such as high thermal and chemical resistance and excellent modulus [11,17,19,20]. Meanwhile, basalt fiber shows a higher tensile strength than E-glass fibers. According to Branston J. et al. [21], it is many times stronger than steel fibers and possesses a few comprehensive qualities, including acid and alkaline resistance [22,23,24]. Therefore, the mechanical properties of basalt fiber reinforced concrete have a comprehensive improvement in flexural strength, spitting strength, modulus of rupture, fracture energy, and abrasion resistance [4,21,25,26,27,28,29,30]. Among the measurements, compression, tension, flexural, and splitting strength have become the main factors in understanding and accessing the fundamental engineering and mechanical properties. After a critical review, the most widely used basalt fiber has a length of 10 to 65 mm with a diameter of 13–20 μm. In addition, the density of basalt is about 1.3 to 2.75 g/cm^3^, the tensile strength is between 2600 to 4840 MPa, and the range of the elastic modulus is 80–115 GPa [31].

Although basalt fiber can be relatively more expensive than some other fibers, it is cheaper than carbon fiber and S-glass fiber. Meanwhile, basalt fiber is more stable in a radioactive environment than synthetic and organic fibers. Since the appearance of related research on the composition of basalt fibers in the 1980s, exhaustive studies of the features and durability of concrete reinforced with basalt fiber have been conducted in the past five years which shows the material’s comparatively good resistance to salt, water, corrosion, and severe degradation in an alkaline environment [32,33].

Moreover, it is globally accepted that chopped basalt fibers can be added to concrete to offset the defects. Therefore, in the past two decades, blending fibers into concrete has been widely applied in some projects [34,35,36,37,38], such as dams, airport sidewalks, and railway sleepers.

### 1.2. The Mechanical Properties of Basalt Fiber Reinforced Concrete

The dosage of basalt fiber and aspect ratio could deeply influence the mechanical behavior of basalt fiber reinforced concrete [39]. Therefore, many researchers have investigated the mechanical properties of basalt fiber reinforced concrete under static conditions.

By studying the impact of the length and volume fraction of basalt fiber on the compressive and flexural strength test under three different lengths and three different dosages, Iyer et al. [40] have further claimed that the optimal length, diameter, and volume fraction under compression and moment of resistance are 36 mm, 16 μm, and 18 kg/m^3^, respectively. According to the pervious study, the performance improvement with the addition of basalt in normal strength concrete or high strength concrete are similar with each other [41]. This shows that the optimal dosage of basalt fiber is 2% with the addition of metakaolin, ranging from 1% to 3% for the compression strength, modulus of elasticity, and split tensile strength.

Following the examination by Highet al. [42] of the usage of dry and procured discrete basalt fiber under the compression and modulus of rupture, Girgin and Yıldırım [43] have also studied the replacement of glass fibers with basalt fibers, showing that basalt fibers are more operational compared to glass fibers under flexural performance. In addition, Hannawi et al.’s [44] examination of the microstructure and mechanical properties of ultrahigh performance concrete with different types of fiber up to 1% shows that basalt fibers can increase the ductility of the specimen, even with the lateral strain, which is the result of its greater aspect ratio than steel fiber, PP-PE fiber, PVA fiber and Barchip fiber.

Furthermore, according to Algin’s [45] study on fresh and hardened concrete with chopped basalt fiber with a length of 12 mm at 0.2%, and 0.4%. 0.6%, and 0.8% of the total volume of concrete mix, the optimization analysis shows that a dosage of 0.356% with a w/c ratio of 0.47 produces the best results.

Table 1 summarises different studies on the impact of dosage on the flexural strength of basalt fiber reinforced concrete in the past decade.

### 1.3. Dynamic Properties of Concrete Properties

Limited studies have been conducted on the dynamic properties of basalt fiber reinforced concrete under dynamic loading conditions such as blast loading, impact response, damping ratio, dynamic modulus of elasticity, and dynamic compression characteristics.

Several studies have recognised moisture content as a primary element of damping capacity. For example, according to Jordan [48], the moisture content in the pore structure will influence the addition of a viscous damping component. Meanwhile, the effect of ageing has also been investigated. In fact, many studies [49,50,51] have suggested that the damping property of concrete decreases as the concrete ages, proving that the interfacial relations of the microstructure within the concrete matrix are the primary property of damping, unlike the compressive strength [48] property.

Meanwhile, the effect of frequency [52] on damping properties has been investigated. In fact, changes existed in the damping properties of the concrete beam under the three main vibrational modes: longitudinal, flexural, and torsional. As a brittle material with various levels of micro-cracks during dynamic bending, the cracks of concrete may cause the matrix to rub on the fiber surface, resulting in energy loss during vibration and a larger damping ratio for fiber reinforced concrete [53,54].

Although studies on the impact of adding steel fiber on the dynamic properties of fiber reinforced concrete show that the damping property of wet-cured fiber reinforced concrete is over 50% greater than plain concrete, the dynamic modulus of elasticity stays relatively constant. Furthermore, ageing and drying can significantly decrease the logarithmic decrement on both fiber reinforced concrete and plain concrete [55]. In addition, as shown in related research [56] on the relation between the damping ratio and other concrete matrix properties the damping ratio may be leveraged as an indication of changing concrete mechanical properties, such as flexural strength, elastic modulus, and ductility. These damping tests are usually advantageous because of their general non-destructive characteristics, meaning that the structure, element, or specimen is unharmed afterwards.

According to Zhang et al. [57],the impact of basalt fiber at various strain rates on the mechanical property of basalt fiber reinforced concrete under a dynamic load, the volume fraction of basalt fiber can vary from 1.325 to 6.625 kg/m^3^. Meanwhile, the concrete specimen is also found to behave in a notably ductile manner due to the increased toughness with the strain rate. In addition, basalt fiber has been tested to optimize the micropore, which is also suitable for high-grade concrete.

After exploring the dynamic properties of basalt fiber reinforced concrete with various fiber volume fractions (1~3%) under different temperatures, Ren et al. [58] have concluded that the behavior of concrete specimens changes weakly under the impact loading due to temperature changes.

Furthermore, according to the micro studies of Fu et al. [59], basalt fibers perform better than polypropylene fibers in stopping fractures due to their increased stiffness. As a result of the more vital relationships between the matrixes, there is no notable change in the surface of the fiber. Meanwhile, some studies, such as those of Fu et al. and Feng et al. [59,60] on basalt fiber reinforced concrete under blast loading show that basalt rebars performed better than steel rebars on many occasions, especially at higher reinforcing ratios. As basalt fiber is far more resistant to blast forces than steel rebars, it can resist explosion energy far better than steel rebars. In this way, the damage degree has been threshold spall at the reinforcement ratio of 1.8%, indicating that micro cracks have occurred followed by hollow sounds and considerable bulges in the concrete, scattering a few fragments on the floor.

The amplitude and frequency content of the excitation source [61] can greatly impact the vibrations in a structure generated by such dynamic loads. Hence, the dynamic properties of concrete materials play a vital role in reducing noise and vibration.

However, the dynamic properties of basalt fiber reinforced concrete have not been identified in the literature, and limited studies have been conducted on combining the residual flexural performance and dynamic properties of fiber reinforced concrete. Therefore, this unprecedented study will primarily explore the performance of basalt fiber-reinforced concrete on residual mechanical properties with the variation of fiber dosage and its dynamic modulus of elasticity test. Moreover, the micro-crack in the real construction work can hardly be identified and some advanced non-destructive testing will be expensive. However, the micro-cracks will be sensitive to vibrations, and the natural frequency is a key parameter to describe the vibrations in concrete. In that case, using the model test to get the natural frequency calculating the dynamic modulus of basalt fiber reinforced concrete in real construction, then comparing the results with the dynamic modulus proposed in this study, is beneficial for engineers to justify the location and number of micro cracks. In this context, this study is the world’s first to identify the dynamic properties of basalt fiber reinforced concrete and the potential relationship between the dynamic and mechanical properties by a series of experimental studies. The new dynamic property tests of basalt fiber reinforced concrete are essential to further clarify the performance of basalt fiber reinforced concrete in shock absorption and sound insulation, and is beneficial to the development of new non-destructive testing.

## 2. Materials and Methods

### 2.1. Material

The concrete specimens in this experiment are all made with Portland cement, well-graded aggregates, water, and basalt fiber [62]. Portland cement (Normal Cem II A-L 42.5 N) [63] was adopted from O’Brien Cement Ltd. in Waterford, Ireland, up to European cement standard I.S. EN197-1:2011. The coarse and fine aggregate materials are sand, gravel, granite, gritstone, crushed rock with quartz, and limestone, all manufactured by Tarmac Trupak of Plumtree Farm Industrial Estate in Birmingham, UK.

The alkaline-resistant basalt fiber is adopted from Deutsche Basalt Faser GmbH (DBF), in which the basalt is chopped to the desired length under a high temperature. This type of fiber also known as integral fibers, such chopped alkaline-resistant fibers have been tested to show no damage when exposed to an alkaline environment at 40 °C and a pH value of 13.5 for 365 days according to the description of Institute of Construction Materials of the Technical University of Dresden. Furthermore, they also present better adhesion performance in concrete due to their special coating. In addition, as shown in Table 2, which shows the mechanical properties of basalt fiber, alkaline-resistant basalt fiber has many advantages compared to other types of fiber. For example, these fibers are not moisture-absorbent compared to mineral wool. Meanwhile, they are not toxic according to DIN 102. They are equipped with a high density at a low insulation thickness of 8 mm and low thermal conductivity. Most importantly, all these characteristics have made them more environmentally friendly than others.

### 2.2. Concrete Mixes

Five concrete mixes have been designed for this study with a water-to-cement (w/c) ratio of 0.55. For all the mixes, the proportions of each concrete mix are 1: 3.1: 2.25 by the mass of cement, fine aggregate, and coarse aggregate, with the details of each concrete mix shown in Table 3.

The coarse aggregate has been prepared under full washing in case of sand’s adhesion on the surface. In addition, both coarse and fine aggregates are dried at the temperature of 105 °C for 48 h.

As demonstrated in a review of previous literature, limited studies on the mechanical properties of basalt fiber-reinforced concrete have shown that the optimal dosage of the basalt fiber roughly ranges within 0.5% of the total concrete volume. Hence, four different dosages are used for each type of basalt fiber reinforcement, ranging from a low dosage to the maximum mixable dosage. In addition, the chopped basalt fiber is added at 0.125%, 0.25%, 0.375%, and 0.5% of content by the total volume of the concrete mix. Finally, the concrete mixes were labelled as M_0_ (plain concrete), M_1_ (0.125%), M_2_ (0.25%), M_3_ (0.375%), and M_4_ (0.5%).

According to the standard test method for fundamental transverse, longitudinal, and torsional resonant frequencies of concrete specimens (ASTM C215-19), to test the hardened concrete, two sizes of concrete specimen have been prepared for compression testing (Cube: 150 mm × 150 mm × 150 mm). This study employs the modal test and the three-points load bending test (rectangular prism: 150 mm × 150 mm × 600 mm), while three beams and five cubes have been made for each mix to ensure enough sample size and avoid errors. Hence, each concrete specimen is labelled as M_x_C/B_x_. For example, M_1_C_2_ means that the concrete specimen is the second cube containing 0.125% of basalt fiber reinforcement. Similarly, M_2_B_3_ shows that the concrete specimen is the third beam containing 0.5% of basalt fiber reinforcement.

### 2.3. Experimental Setup

Some relevant tests in the laboratory have been conducted, including the slump test (BS EN 12350-2:2019), the compression test (BS EN 12390-3:2019), the residual flexural strength test (BS EN 14651:2005), and the dynamic modal test (ASTM C215-19).

**Figure 1:**
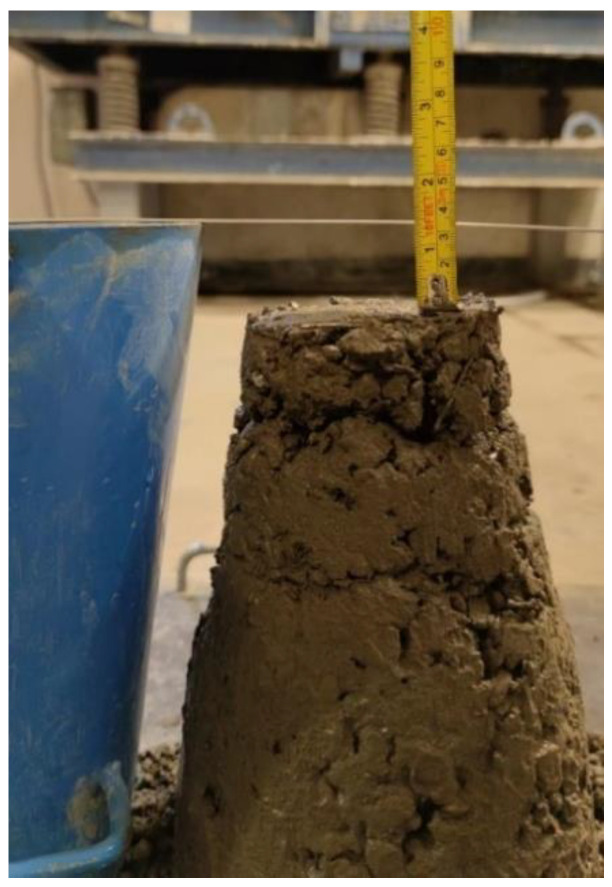
Slump test of the M_3_ concrete mix.

In these tests, the components have been placed in the remixing container in the following order: aggregates, cement, basalt fiber, and water. When the cone and base plate are dampened and the excessive moisture with the moist cloth is removed, the concrete matrix is filled to the slump retention at a specific time with three layers compacted with 25 strokes of the compacting rod, respectively. The cone is then raised for 2 to 5 s to avoid the lateral or torsional motion formed on the concrete. By measuring the heights of the cone and the highest point of the slumped test specimen, the slump h can be calculated and recorded, as shown in Figure 1.

When the mixing of the concrete matrix is done, it is cast in a mold with the sizes of 150 × 150 × 150 mm^3^ (cube) and 150 × 150 × 600 mm^3^ (rectangular prism). Moreover, the 5-mm-wide notch is placed on one side of the cuboid mold for a three-point bending test of a single-side notched beam on the specimens. Finally, the specimens are demoulded after 48 h and cured under the water for at least 28 days.

The compressive strength of the cube specimens has then been tested under a universal testing machine from Norton Hydraulics (Midland) in Birmingham, UK at the rate of 0.5 MPa/s. In fact, the flexural performance of the beam specimen can be determined by the load-displacement curve obtained by simply testing the supported beam specimens and load-CMOD (Crack Mouth Opening Displacement) curve obtained by the Crack Opening Displacement (COD) gauges.

**Figure 2:**
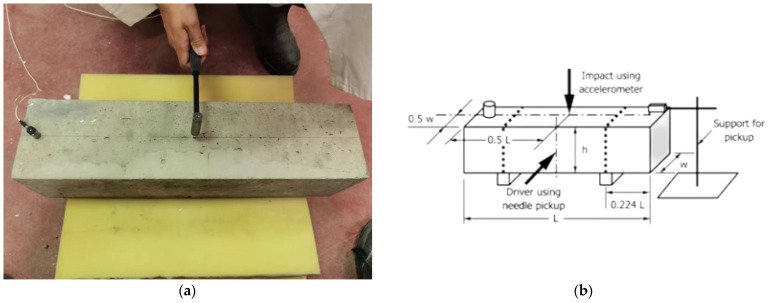
Modal test of the concrete specimen (ASTM C215-19). (**a**) The experiment setting; (**b**) details of the equipment setting.

Furthermore, the resonant frequencies of the dynamic properties of specimens can be obtained from the detected driving force with a hammer, which is shown in Figure 2. Meanwhile, the amplifiers are adjusted in the driving and pickup circuits to provide a noticeable indication. As the driver is positioned, the driving force can be perpendicular to the specimen surfaces when the specimens are supported for free vibration in the transverse mode. In this way, the specimen will be forced to vibrate at different frequencies, while the different indications of the amplified pickup outputs are recorded.

Lastly, the modal test is normally set ahead of the three-point loading test because it causes no damage to the specimen. In addition, the mass and dimensions of the specimen are measured to calculate the dynamic MOE (modulus of elasticity) from Equation (1) in ASTM C215-19.

(1) $$\mathrm{Dynamic}\;E=CMn^{2}$$

M = mass of the specimen, kg,

*n* = fundamental transverse frequency, Hz,

C = 0.9464 (L^3^T/dt^3^), m^−1^ for a prism,

*d* = diameter of cylinder, m,

*t* = dimension of prism cross section in the direction the prism is driven or impacted, m,

*b* = dimension of prism cross section perpendicular to *t*, m,

*T* = correction factor that depends on the radio of the radius of gyration, (This experiment was scored as 1.39).

## 3. Results and Discussion

### 3.1. Fresh Concrete Workability

According to Table 4, which lists the slump of the basalt fiber-reinforced concrete, the slump has slightly dropped with the increasing dosage of basalt fiber up to 0.5%, which peaked at 43 mm in the plain concrete matrix M_0_. However, when adding basalt fiber with the volume fraction of 0.125%, 0.25%, 0.375% and 0.5%, the slumps of the concrete matrix has fell to 40 mm, 33 mm, 26 mm, and 18 mm, respectively. Therefore, the addition of fibers in concrete can result in a reduction in the workability of the concrete. In this way, the dispersed basalt fiber in the concrete, which might form a net-shaped structure, makes this phenomenon possible because it has prevented the mixture’s segregation and flow. According to the degree of workability suggested in the standard, the range of slump is 25–50 mm, which means that the workability of concrete is low and the slump between 0–25 mm is very low. It can be seen that the addition of basalt fiber led to a decrease in the workability of concrete, and the level of workability dropped to very low workability with a fiber content of 0.5%. Due to the fiber’s high content and surface area, it may absorb more cement paste to wrap around the fiber itself, making the mixture more viscous while reducing the slump [64].

### 3.2. Mechanical Property Test

#### 3.2.1. Compression Test

According to Table 4, which presents the results of the concrete cube specimen’s compression after 28 days, the compressive strength of all the mixes does not show a conspicuous change as the increment varies in the small zone while forming a slight trend. Firstly, the results of five specimens from the same patch has shown stable properties as the standard deviation value ranges within 1.00. Secondly, the trend has grown to 43.684 MPa with a 0.25% basalt fiber addition, before going down flatly to 41.5 MPa with a 0.5% basalt fiber addition. In addition, when it comes to M_4_, the value of the 0.5% volume fraction appears to be flat on the M_1_ (0.125%). However, compared to plain concrete, certain improvements can still be achieved.

**Figure 3:**
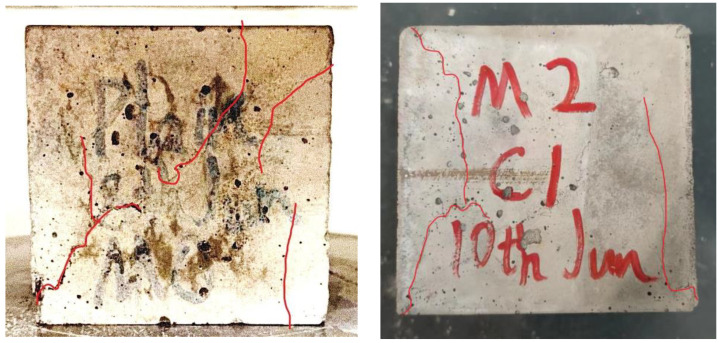
Cracks of the specimen after the compression test by contrast. (Left is cube of M0, and right is cube of M2).

**Figure 4:**
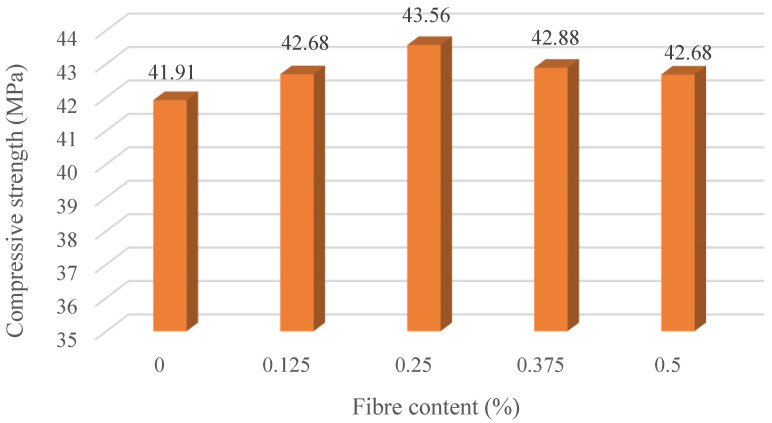
Compressive strength under different mixes.

According to Figure 3, the plain concrete specimen seems to have developed more longitudinal cracks than specimens with added basalt fiber, because the cracks on the longitudinal cross-section of M_2_ are shorter than plain concrete specimens after the compressive loading. In other words, the distribution of cracks is considered more dispersed with the increasing fiber in the concrete specimen. Figure 4 portrays the influence of fiber addition on the compressive strength of basalt fiber reinforced concrete.

Besides, a previous Branston study [22] shows that there might be a monotonical decrease with the increment of fiber. The applied basalt fiber is 36 mm and 50 mm. Compared to its irregular result, the shorter length of the basalt fiber seems to show more details to test the impact of the fiber dosage on the compressive performance of the basalt fiber-reinforced concrete. In addition, the fiber-bridging force existing in fiber-reinforced concrete can delay the macro crack propagation while restricting the development and opening of the macro cracks [28]. Moreover, such a behaviour also helps to prevent the concrete from lateral expansion by holding back the fracture formation. Therefore, although adding more basalt fibers to concrete cannot reinforce its peak compressive resistance, a gradual decrease in the load capacity and the enhancement of structural integrity can be seen. In this way, the long and chopped fibre can cause conjunction during the mixing, resulting in blockages and clumps in concrete.

#### 3.2.2. Flexural Performance

Table 5 lists the experimental results of the basalt fiber-reinforced concrete beam specimens. The values of the proportionality (LOP) presented as *F_fc,t_,* the span length shown as *L*, and the residual flexural tensile strengths referred to as *F_R,1_, F_R,2_, F_R,3_*, and *F_R,4_* listed in Table 5 are the averages of the three beam specimens per test variable. Therefore, the residual flexural strength has been calculated with the following Equation (2), which is defined in the BS EN 14651: 12.

(2) $$f_{R,j}=\frac{3P_{j}L}{2bh_{s_{P}}^{2}}$$

L is the span length, mm;

$P_{j}\;$is the applied load, N;

b is the width of the specimen, mm;

$h_{s_{P}}$ is the distance between the tip of the notch and the top of the specimen, mm.

Where$\;f_{R,j}$ and $P_{j}$ are the residual flexural tensile strength and the applied load, respectively, which correspond to CMOD_j_. In this way, *F_R,1_*, *F_R,2_*, *F_R,3,_* and *F_R,4_* are determined by the corresponding CMOD values of 0.5 mm, 1.5 mm, 2.5 mm, and 3.5 mm, respectively.

The results of the three specimens of each mix were obtained under the there-point load bending test. Each median result of the three specimens has been taken as a reference for a more concise analysis on flexural performance considering the results of each mix varied in a small proportion, as shown in Table 5.

The continuous growth of flexural strength can be observed to exist with the increasing dosage of basalt fiber in the concrete specimens. Meanwhile, the LOP (f_ct_, L) of the plain concrete specimen stays at 3.49 Mpa with the peak load of 9.078 kN. With the increasing volume fraction of basalt fiber from 0.125% to 0.5%, the increment amount ranges from approximately 4.15% to 46.85%. In addition, the displacement at the peak load is shorter and earlier than plain concrete when more fiber volume fractions are added. Therefore, the increment has shown a uniform growth curve, with M_4_ (0.5%) having the peak load of 13.331 kN and the fastest speed to reach it.

**Figure 5:**
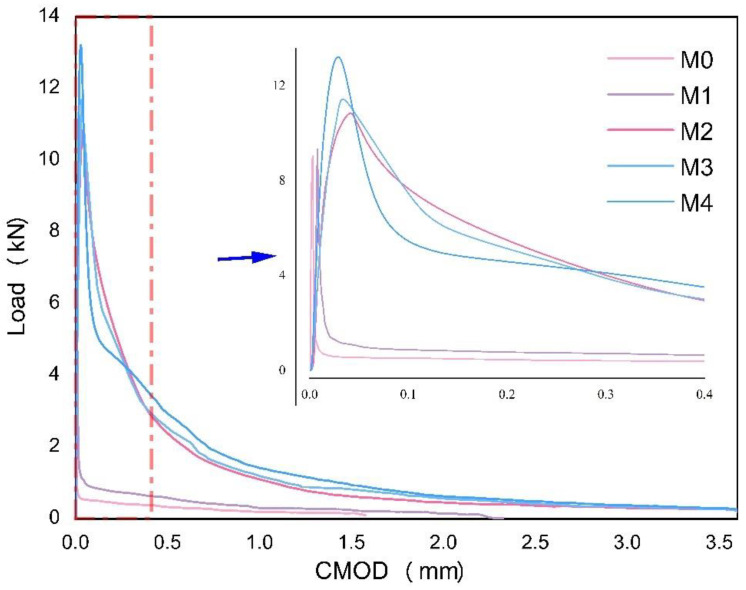
The Load-CMOD curve of the bending test (3.5 mm and 1.0 mm).

From Figure 5, it can be seen that the residual flexural tensile strength is linearly proportional to the CMOD before it reaches LOP, generally forming a relative elastic zone. However, the curve is abruptly shifted once it reaches the LOP. Meanwhile, the peak load value shows a trend of increase with basalt fibers in concrete, indicating that basalt fiber-reinforced concrete can improve the initiation of tensile cracks. On the other hand, once the first peak or cracking strength occurs, the flexural tensile strength sees a sudden decrease. Therefore, the magnitude of the strength reinforcement is mainly dependent on the content ratio of the fibers.

**Figure 6:**
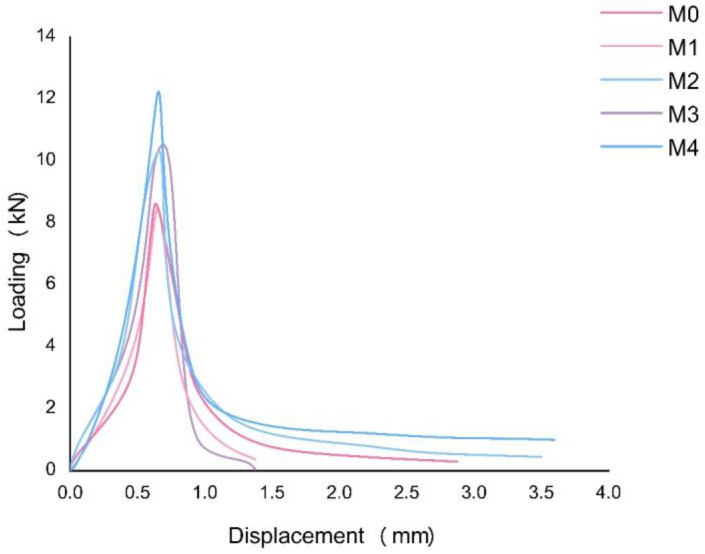
The load-displacement curve of the bending test.

Comparatively, when the curves dropped after the peak, the curves of each mix can be divided into two groups regarding the residual flexural tensile performance, with M_0_ and M_1_ showing a more drastic drop when reaching their peaks. As shown in Figure 6, while the applied load values of M_0_ and M_1_ are 0.21 and 0.29 kN, respectively, those of M_2_, M_3_, and M_4_ are 5.1, 7.0, and 7.4 kN, respectively. Moreover, the two groups have shown different slopes when the CMOD comes to 0.1 mm. The curves of mixes containing less than 0.25% volume fraction of basalt fiber remains at approximately 1 kN. On the other hand, a load of mixes with over 0.25% volume fraction of basalt fiber shows a sign of dropping at the relatively higher level but with a slower slope.

**Figure 7:**
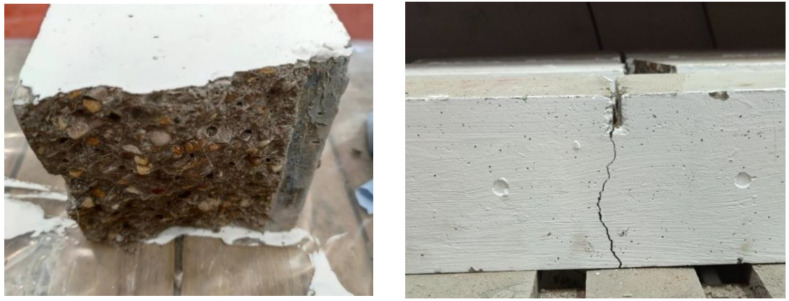

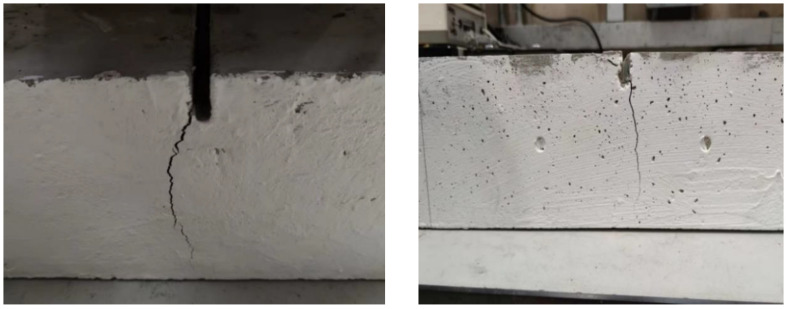
The post-crack of the concrete specimen after the bending test by contrast (left upper is M0, right upper is M1, left bottom is M2 and right bottom is M3).

According to Figure 7, several cracks appeared on the plain concrete specimen when the load increased, causing it to suddenly break into two pieces on the machine. Hence, the load stopped at a CMOD of 1.7 mm. In this way, the M_1_ specimens can usually last longer on the machine than the plain specimens. In addition, the M_1_ is likely to be broken into two pieces when it is removed from the machine. When the volume fraction of basalt fiber is over 0.25%, the load-CMOD curve will have a smooth downward trend until it is over 3.5 mm of the test range. Moreover, the residual flexural tensile strengths of M_2_, M_3_, and M_4_ are higher when more fiber is added. As shown in Table 5, residual flexural tensile strength will increase slightly between M_2_ and M_3_ with the trend being more conspicuous from M_3_ to M_4_. In those cases, the addition of fiber contribution to the increment of residual flexural strength from 0.125% to 0.5% of basalt fiber. Figure 7 shows that there are abundant ruptured basalt fibers on the split cross-section of the beam specimens. The concretes [3] perform the best with high ultimate loads, greater deflections, and higher fracture energy values at the post-peak zone as the basalt fiber content rises. Therefore, the bridging force of the fibers can significantly impact the property of plain concrete from brittleness to ductility.

### 3.3. Modal Testing

#### 3.3.1. Damping Ratio

**Figure 8:**
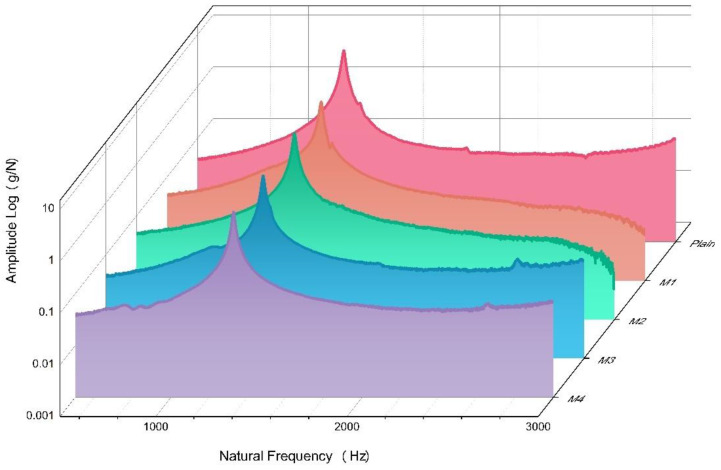
The relationship between amplitude and natural frequency of each mix during the modal test.

The dynamic tests have been carried out above a strong floor with very little noise and vibration in order to avoid the interference of external vibrations. The specimen is placed above two layers of sponge cushion with a total thickness of 10 cm, which aims to provide a free-free condition. To ensure the accuracy of the experiments, they have all been performed by one operator and used a similar excitation force. Ten taps are performed for each experiment, and the FRF curve (see Figure 8) is calculated based on the software. According to Table 6, which shows the natural frequency and damping ratio in transverse mode, the standard deviation value of the damping ratio of each three beams varies from a small range, especially on M_2_ and M_3_, which have shown a high level of consistency. In fact, the fiber-reinforced specimen has a similar damping ratio with plain concrete, which is approximately 0.59%. Due to its variation, changes can be deduced with the addition of basalt fiber. Furthermore, the 0.5% volume fraction of basalt fiber has no obvious impact on the damping ratio of the basalt fiber-reinforced concrete.

#### 3.3.2. Dynamic MOE (Modulus of Elasticity)

The dynamic MOE value of each mix can be calculated with Equation (1). According to the experimental result, the dynamic MOE and the natural frequency present a similar trend when the basalt fiber is added. The natural frequency will increase to 1345.6 Hz when 0.25% of basalt fiber is added to the concrete, showing a dynamic MOE of 31.44 GPa. When considering plain concrete as a benchmark, the increment of dynamic MOE is 13.83% at the peak (0.25%). The dynamic MOE then sees a continuous decline with a slower slope than the rising slope. In comparison, when adding 0.5% of basalt fiber, the dynamic MOE is 29.86 GPa, which is lower than the M2 (0.25%) value. However, compared to plain concrete, it still has an 8.1% increment.

From the results of the modal test, as shown in Table 7, and the compressive test, as presented in Table 4 and Figure 4, the compressive strength and dynamic MOE have shown a similar trend when the volume fraction of basalt fiber is increased continuously, with M_2_ (0.25%) showing the highest compression and dynamic MOE value. In addition, according to the load-displacement curve of the residual flexural tensile strength, M_2_ (0.25%) is the mix that has reached its peak load, which, in another method, can represent the relationship between the strength and strain of the concrete specimens.

From the result of the modal test (Table 7) and compressive test (Table 4), the compressive strength and dynamic MOE showed a similar trend when continuously adding the volume fraction of basalt fiber. M_2_ (0.25%) has the highest compression and dynamic MOE value. In addition, the load-displacement curve of residual flexural tensile strength also showed that M_2_ (0.25%) is the fastest mix to reach its peak load, which, in another method, could display the relationship between the stress and strain of the concrete specimens.

Research [65] shows that the dynamic modulus of elasticity could be correlated to the compressive strength of high-strength self-compacting concrete. Lu et al. [64] stated that both dynamic MOE and static MOE are relevant to compressive resistance. An empirical equation was concluded for estimation purposes. Hence, concrete with higher stiffness/MOE normally has a higher compressive performance.

Furthermore, a previous study [65] also shows that the dynamic modulus of elasticity can be correlated to compressive high-strength self-compacting concrete. For example, according to Lu et al. [66] and Li et al. [67,68,69,70], both dynamic and static MOE are relevant to compressive resistance. Therefore, an empirical equation can be settled for estimating, showing that concrete with higher stiffness/MOE usually has a higher compressive performance.

## 4. Conclusions

Based on the results from a series of experiments, conclusions can be drawn to develop new insights into basalt fiber reinforced concrete for industrial applications.

The workability of fresh concrete elicits signs of decline with the increasing dosage of basalt fiber, with the slump decreasing from 44 mm to 18 mm from plain concrete to concrete, with a volume fraction of 0.5% for basalt fiber. Among concrete with volume fractions of 0.125%, 0.25%, 0.5%, 0.375%, and 0.5% of basalt fiber in plain concrete, the compression of concrete with 0.25% of basalt fiber dosage has the highest performance (43.98 MPa), which is 4.48% higher than that of plain concrete. Meanwhile, the flexural strength (LOP) has greatly improved, showing a monotonical trend of increase when the volume fraction of basalt fiber is increased from 0.125% to 0.5%. The peak flexural strength is 4.15%, 20.61%, 28.70%, and 46.85%, respectively. Furthermore, the residual flexural tensile strength shows a smooth descending CMOD-load curve after the peak when the basalt fiber content is above 0.25%. In addition, the damping ratio remains around 0.60, which ranges from plain concrete to a volume fraction of 0.5% for basalt fiber. Given the results of the experimental study, these new conclusions can be drawn:

- The addition of basalt reduces the workability of concrete significantly
- The compressive strength of basalt fiber concrete does not increase with the addition of fiber, and the suggested fiber content is 0.25%
- The flexural strength increases sharply with the addition of basalt fiber when keeping the fiber content lower than 0.5%
- The MOE of basalt fiber concrete is affected by the compressive strength, and there is no significant impact on the damping ratio of concrete with the addition of basalt fiber.

This study is the first to demonstrate the essential parameters of mechanical and dynamic properties of basalt fiber concrete through experimental studies. Based on these new insights, engineers can evaluate the performance of their basalt fiber concrete structures, especially in aggressive environments. In addition, the dynamic moduli and damping coefficients reported in this study can be applied for dynamic structural designs and the non-destructive testing of basalt fiber reinforced concrete structures.

## Figures and Tables

**Table 1:** Research status of the optimal dosage of basalt fiber for flexural performance.

Scholar	Test Methodology	Flexural Properties	Dosage (%)	V_opt_ (%)	Increment (%)
Kabay [3]	Three-Point Bending Test on Notched Beams	Flexural Stress	0.07, 0.14	0.14	13
Jiang [19]	Australian Standard, As1012.11-1985	Strength-Effectiveness and Flexural stress	0.05, 0.1, 0.3	0.3	25.51
Jun [46]	Three-Point Bending Test	Flexural Stress	0.1, 0.15, 0.2, 0.25, 0.3, 0.35	0.3	12.3
Arslan [25]	Three-Point Bending Test on Notched Beams	Flexural Stress	0.021, 0.042, 0.084, 0.126	0.084	25.37
Branston [21]	Astm C1609	Residual Flexural Stress	0.15, 0.31, 0.46	0.46	180
Jalasutram [27]	Astm C1609	Residual Flexural Stress	0.5, 0.1, 0.15, 0.2	0.2	80
Katkhuda [47]	Three-Point Bending Test	Flexural Stress	0.1, 0.3, 0.5, 1, 1.5	1.5	74
Zhou [28]	Three-Point Bending Test	Flexural Stress	0.1, 0.2, 0.3, 0.4, 0.5, 0.6	0.3~0.4	40

**Table 2:** Parameter of basalt fiber in the study.

Basalt Type	Short-chopped Filament	Length [mm]	25
Single Diameter [μ]	13~17	Temperature Resistance [°C]	750
Density [kg/m^3^]	2800	Thermal Conductivity [W/mK]	0.031
Areal Density [kg/m^2^]	1	Tensile Strength [MPa]	4840
Width × Run Length [mm]	1 × 10	Elastic Modulus (GPa)	89
Absorption of Humidity (%)	≤0.1	Elongation (%)	3.15

**Table 3:** Proportion of concrete mix design for 1 m^3.^

Materials	Cement	Gravel	Sand	Water
Weight (kg/m^3^)	317.3	713.925	983.63	174.515

**Table 4:** The result of the compression and slump test.

Mix	Slump (mm)	Compressive Strength (MPa) 28 Days						
		1	2	3	4	5	* f_c_ ± SD (MPa)	Increment (%)
Plain	43	42.02	40.25	43.08	42.06	42.06	41.91 ± 1.03	42.06
0.125%	40	41.22	42.41	44.26	42.99	42.99	42.68 ± 1.10	42.99
0.25%	33	41.78	44.01	43.97	43.98	43.98	43.56 ± 1.10	43.98
0.375%	26	43.17	42.21	43.56	42.51	42.51	42.88 ± 0.53	42.51
0.5%	18	42.98	42.87	43.11	42.92	42.92	42.68 ± 0.66	42.92

* f_c_ is the compressive strength and SD is the standard deviation.

**Table 5:** The result of residual flexural strength.

Flexural Strength (MPa) 28 Days					
Mix	Plain	0.125%	0.25%	0.375%	0.5%
Peak Load (kN)	9.078	9.455	10.949	11.684	13.331
Increment (%)		4.15%	20.61%	28.70%	46.85%
Displacement (mm)	0.003	0.055	0.042	0.032	0.028
fct, L	3.486	3.631	4.204	4.487	5.119
FR, 1	0.127	0.865	0.923	0.956	1.132
FR, 2	0.061	0.114	0.238	0.319	0.356
FR, 3	0.000	0.063	0.141	0.166	0.188
FR, 4	0.000	0.000	0.103	0.104	0.113

**Table 6:** The result of the modal test.

Concrete	Mix	Natural Frequency (Hz)			Damping Ratio		
		Sample Value	Average	SD	Sample Value	Average	SD
Plain	M_0_B_1_	1267.2	1265.3	1.36	0.50%	0.56%	0.052
	M_0_B_2_	1264.1			0.55%		
	M_0_B_3_	1264.6			0.62%		
0.125%	M_1_B_1_	1301.9	1298.9	2.10	0.64%	0.60%	0.033
	M_1_B_2_	1297.4			0.57%		
	M_1_B_3_	1297.5			0.57%		
0.25%	M_2_B_1_	1347.4	1345.6	1.34	0.60%	0.60%	0.003
	M_2_B_2_	1345.2			0.59%		
	M_2_B_3_	1344.2			0.60%		
0.375%	M_3_B_1_	1327.9	1323.967	2.79	0.58%	0.59%	0.008
	M_3_B_2_	1321.8			0.60%		
	M_3_B_3_	1322.2			0.59%		
0.5%	M_4_B_1_	1321.2	1322.167	0.97	0.61%	0.58%	0.021
	M_4_B_2_	1321.8			0.56%		
	M_4_B_3_	1323.5			0.57%		

**Table 7:** Dynamic MOE of each mix in the modal test.

	Density of Beams (kg/m^3^)	Natural Frequency [Hz ± SD]	Damping Ratio [% ± SD]	Dynamic—MOE [GPa ± SD]
Plain	2277.04	1265.3 ± 1.36	0.56 ± 0.052	27.62 ± 0.06
0.125%	2264.44	1298.8 ± 2.10	0.60 ± 0.033	29.06 ± 0.09
0.25%	2275.56	1345.6 ± 1.34	0.60 ± 0.003	31.44 ± 0.06
0.375%	2282.22	1324.0 ± 2.79	0.59 ± 0.008	30.05 ± 0.13
0.5%	2276.30	1322.2 ± 0.97	0.58 ± 0.0021	29.86 ± 0.04

## Data Availability

Data can be made available upon reasonable request to the corresponding author.
